# Differences in Interactions with a Conversational Agent

**DOI:** 10.3390/ijerph17093189

**Published:** 2020-05-04

**Authors:** Young Hoon Oh, Kyungjin Chung, Da Young Ju

**Affiliations:** 1 School of Integrated Technology, Yonsei Institute of Convergence Technology, Yonsei University, 85 Songdogwahak-ro, Yeonsu-gu, Incheon 21983, Korea; 50hoon@yonsei.ac.kr; 2 Design Intelligence, School of Communication & Arts, Yonsei University, 85 Songdogwahak-ro, Yeonsu-gu, Incheon 21983, Korea; kjchung495@yonsei.ac.kr; 3 Department of Future Design, Graduate School of Techno Design, Kookmin University, 77 Jeongneung-ro, Seongbuk-gu, Seoul 02707, Korea

**Keywords:** conversational agent, older adult, human-agent interaction

## Abstract

Recent technological advances introduced conversational agents into homes. Many researchers have investigated how people utilize and perceive them. However, only a small number of studies have focused on how older adults interact with these agents. This study presents a 14-day user study of 19 participants who experienced a conversational agent in a real-life environment. We grouped them into two groups by age and compared their experiences. From a log study and semi-structured interviews, we identified several differences between the two groups. Compared to younger adults, older adults used the agent more. They used it primarily for listening to music and reported satisfaction with it. Younger adults mainly used utility skills like weather report checks and setting of alarms, which streamlined their daily lives. Moreover, older adults tended to view the agent as a companion, while younger adults saw it as a tool. Based on these empirical findings, we suggest that conversational agents should be designed with consideration of the different usage patterns and perceptions across age groups.

## 1. Introduction

Conversational agents (CAs), which are able to recognize verbal input and generate verbal output [1,2,3,4], are becoming integrated into our lives. The emergence of CAs such as Apple’s Siri and Google’s Google Now has attracted industry as well as academia. Recent technological advances have placed CAs in homes in the form of voice-controlled speakers. The most representative example is Amazon’s Echo, which is widely available on the market [5]. Thanks to its rapid distribution, many human-agent interaction (HAI) researchers have utilized it.

The HAI community has studied not only CA usage but also user perception of it. They found that some people treat CAs as a person, not just a task-oriented machine [6,7,8]. Some users tended to personify the CA [6,8,9,10], and this was linked to higher satisfaction with it [10]. It is known that the perceptions of CAs may change over time [11].

Recent studies have reported that CAs provide social support and various psychological benefits to the elderly [12,13,14,15,16,17]. They have also reported that they can reduce loneliness in the elderly [18]. However, previous studies have not investigated the characteristics of the elderly in the HAI in-depth. The elderly can benefit from a CA, and understanding their perceptions of it is an important research area [19]. In this regard, this paper presents several differences between younger adults and older adults’ interactions with Clova, which is a voice-controlled smart speaker similar to Amazon’s Echo. We analyzed 14-day CA usage logs [7] and semi-structured interviews conducted with 19 participants. Our study results showed that the type of Clova usage differed by age group. The elderly listened to music significantly more than the younger adults did, while the younger adults extensively used the utility skills that they need every day, such as weather reports or setting an alarm. Moreover, interviews indicated different perceptions toward Clova; the elderly described Clova as a companion and showed high levels of personification of it, while younger adults viewed it as a useful tool to facilitate living efficiently.

## 2. Related Works

### 2.1. Interactions with a Conversational Agent

With the emergence of commercially available CAs, the HAI community has studied how they could be integrated into our lives. To understand how people interact with CAs, researchers investigated their usage using a diary [8,20], survey [21], and interview [7,9,11,20]. Other researchers analyzed online reviews to investigate the perceived roles and preferred features of CAs [6,10,13]. These studies show that users mainly use CAs for checking the weather or listening to music [7,8,9,22]. Hands-free voice interaction was the motivating rationale behind their interactions [6,23]. Enabling multitasking facilitated its use [9], and the ease of speech input lowered the hurdles for them to get the desired results. This resulted in a positive attitude toward CAs [6,10,24].

Beyond self-reported data, a few studies have investigated the CA usage log. The authors of [24] conducted a pioneering study in which they analyzed the logs of Alexa voice commands. Based on more than 200,000 logs, the researchers presented 24-h timelines to show the context of CA usage. Checking weather was primarily used in the morning, whereas smart home commands and timer settings were executed at night. The music command was used regardless of the time of day. These trends were observed from Google Home users as well [22]. Moreover, Cho et al. investigated the long-term usage of Alexa [11]. They focused on how user experience has changed over time. They found that Alexa users lost interest in the agent and showed minimal usage over time. In summary, a few CA studies analyzed the voice command logs and identified the context of its use. However, only a small number of studies focused on the age-related differences in perception of CA [25]. An understanding of the elderly’s CA usage is missing from the literature [19,26], and it is the key contribution of this paper.

### 2.2. Personification of Conversational Agent

Although the communication capability of CA needs significant improvement [9,27], some studies reported that users tend to personify it. Turk reported that CA users frequently used personifying expressions such as “thank you” or “good morning” to CAs [28]. Similar findings were reported from other studies as well. Although they were not based on direct observation of CA usage, Purington et al. [10] demonstrated the existence of personification and its effect on HAI. The more people used personal pronouns toward Alexa, the more social interactions with “her” were observed. In addition, this personification behavior was linked to higher satisfaction with the CA [8,10]. Inspired by [10], Gao et al. conducted a larger-scale study of sentiment analysis on Amazon Echo reviews and found similar results; many customers regarded Alexa as a friend, family, or even a girlfriend [6]. These studies indicate that CAs are perceived not only as typical electronic machines but also as companions [13,20,29,30].

## 3. Method

### 3.1. Participants

Initially, 20 participants were recruited through word of mouth. However, one older participant (P15) withdrew from the experiment because her travel schedule overlapped with the study period. This left us with 19 participants in the study (mean age = 51.74 years, Table 1). The study was conducted in their homes because Clova requires a wireless network connection. To investigate the differences in the CA use by age group, we divided participants into two groups: older and younger [31]. For this paper, the older group included all participants aged 50 years and older, and the younger group comprised participants aged under 50 years old [31,32,33]. After the end of the experiment, all participants were paid about $300 for participating in the study.

### 3.2. Apparatus

Alexa has been widely adopted in many CA studies, but it does not officially support Korean language. For this reason, we used Clova for this study. Clova is a representative artificial intelligence (AI)-powered CA that supports Korean [29,34,35]. Clova is also the wake word of Clova-enabled smart speakers. Similar to Amazon’s Alexa, Clova recognizes the user’s voice, uses a female voice, and has daily conversations with users. It performs various skills such as playing music, checking the weather, and setting alarms (Table 2). Similar to other voice-controlled speakers, it has a mute button, volume up/down buttons, Bluetooth pairing button, and power port on the back.

**Figure 1:**
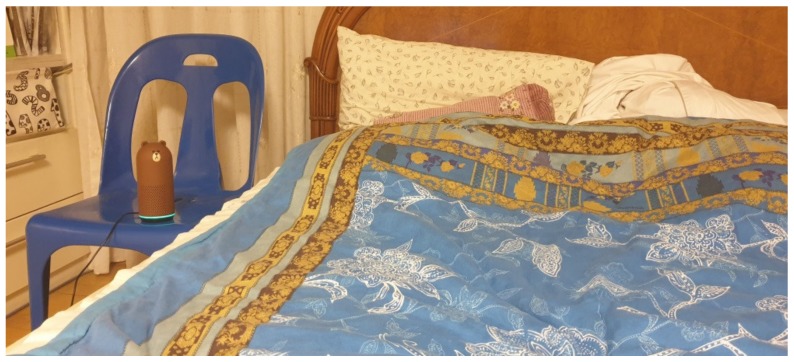
Conversational agent Clova with its green LED turned on.

Furthermore, using Clova is similar to using other voice-controlled speakers such as Amazon’s Echo or Google Home. If the agent recognizes the wake word, it switches on the green LEDs at the bottom of the speaker (see Figure 1). The users can give voice commands while the green light is on. For instance, if a user says “Clova,” (green light turns on), “play classical music,” then Clova acknowledges the user’s command (“Yes, I’ll play classical music from Vibe” [36]) and streams classical music from the music service provider [36]. In addition, the user’s voice commands and Clova’s responses are recorded in the companion mobile application. If Clova played music, the name of the artist and the title of the song are recorded. If the user checked the weather, the text with which Clova verbally responded is recorded.

### 3.3. Procedure

Two researchers visited each participant’s home and explained the purpose and duration of the study. Then they introduced Clova to the participants and set it in a location chosen by the participants (mostly in the living room or bedroom). One Clova was distributed to each household. In addition, Clova’s mobile companion application was installed on the participant’s phone, and they logged in with a separate account prepared for the study.

After Clova was installed, the participants were given detailed instructions on how to use it. Researchers demonstrated several skills of Clova, which are listed in the official Clova user manual, such as “Clova, how’s the weather today?”. The participants were encouraged to test voice commands, and they asked the researchers questions about its features. To prevent them from forgetting how to use it, we handed out user manuals that contained the instructions we explained, such as the wake word and the steps to a command. The participants were also instructed on how to delete the conversation logs from the Clova smartphone application if they did not want to share them [24].

We surveyed their demographic information after the instructions were completed. All participants gave informed consent and volunteered to participate in the study. The study was performed in compliance with the ethical recommendations of the Declaration of Helsinki. After the end of the experiment, we re-visited their homes and conducted semi-structured interviews (interview length mean = 44.55 min). In the interview, they were asked to (a) describe frequently used features and the context in which they interacted with Clova; (b) explain their satisfactory or unsatisfactory experience; and (c) discuss improvements and expectations of Clova. All interviews were audio-recorded with the consent of the subjects and transcribed verbatim.

### 3.4. Data Analysis

We manually backed up the 14-day voice command log recorded in the Clova application. To collect the log data, we logged into the Clova application from another phone used exclusively for research. We logged in with the separate account that was used to set Clova up at the participant’s house and backed up the voice commands that were shown in the mobile application. The collected log data were speech-to-text results for the participant’s speech, Clova’s response, and the date of the voice command. The time of the command was not recorded because it is not supported by the application. Then we categorized the logs referring to the official guide of Clova voice commands [35]. For instance, weather checking was categorized as lifestyle information (see Table 2).

While categorizing the data, we observed many incomplete interactions (e.g., speech recognition failure and executing unsupported feature), so we coded whether an interaction was successful or not. Even when participants spoke correctly, there were cases in which the agent did not provide the desired results or did not recognize their voice. Therefore, we additionally coded the interaction logs as follows: the number of interactions that were successful (hereinafter referred to as total successful interactions), the number of interactions that failed (hereinafter referred to as total failed interactions), and the total number of interactions. The total number of interactions is the sum of total successful interactions and total failed interactions. Two researchers conducted the coding and another researcher verified the coded results. The criteria for the coding were as follows:

- Cases categorized as successful interaction: Clova correctly recognized the user’s voice and provided the expected response.
- Cases categorized as failed interaction: (i) Clova correctly recognized the user’s voice but failed to provide the expected response; (ii) Clova did not recognize the user’s voice correctly and failed to provide the expected response.

Table 2 shows the voice command categories that were analyzed in this study. The categories and the examples are described in [35] and were translated to English by a professional translation agency.

#### 3.4.1. Clova Usage Log Analysis

We analyzed the Clova usage logs of the 13 households and investigated whether their usage differed by age group. We normalized the log data of each voice command category according to the age group. After normalization, we conducted the normality tests on the data. For the majority of the voice command categories, the Shapiro–Wilk test showed statistically significant results (*p* < 0.05). Therefore, the data did not fit a normal distribution, and we performed the Mann–Whitney U tests to compare the CA usage between the older and younger adults. All statistical analyses were completed using SPSS version 25 (IBM Corp, New York, NY, USA) and G*Power 3 (Heinrich-Heine-Universität Düsseldorf, Düsseldorf, Germany) [37]. The statistical hypotheses we evaluated were as follows:

1. **H_1_:***The mean proportions of the total successful interactions and total failed interactions per household will differ by age groups.*
2. **H_2_:***The mean proportion of the Clova usage per household for each of the specified voice command categories will differ by age groups.*

#### 3.4.2. Qualitative Analysis

Recorded interviews were transcribed prior to analysis. The interviews were analyzed with an inductive thematic analytic approach [38]. Two researchers conducted the qualitative analysis independently. They familiarized themselves with the data by repeatedly reading the entire transcripts. Then they generated initial codes, identified several patterns from the interviews, and discussed their results to reach a consensus about the generated themes. After converging and comparing the emerging themes, they were grouped by the participants’ perceptions and voice command categories of Clova. Some representative quotations were used to support the findings.

## 4. Findings from the Log Study

### 4.1. Overall Usage

**Figure 2:**
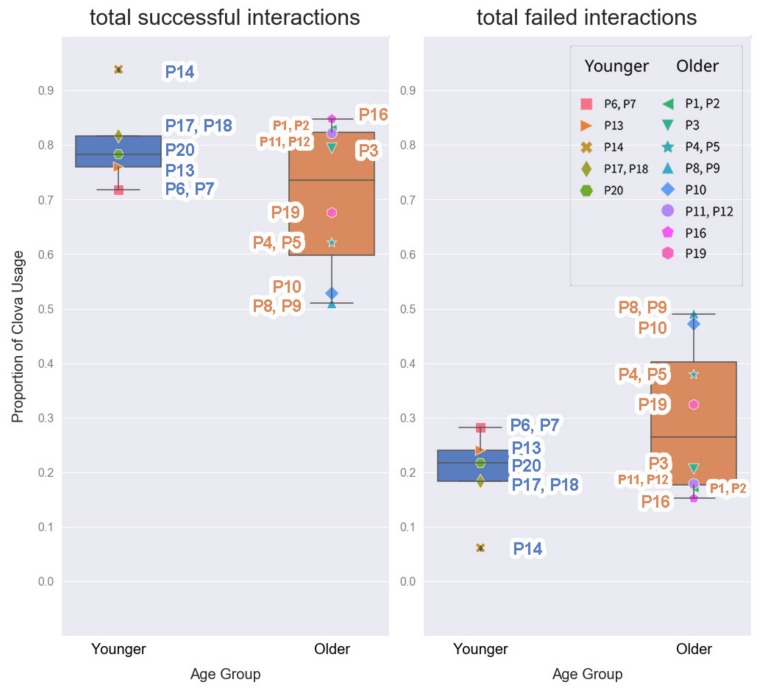
Proportion of Clova usage by age group: total successful interactions and total failed interactions.

We remotely gathered and analyzed 1600 voice commands from the 14-day experiment. The Mann–Whitney U tests found several significant differences in CA usage: music/audio, lifestyle information, and schedule management. The total number of interactions of older adults was higher than that of the younger group (Table 3). The older adults’ high CA usage may be due to the long time they spent at home. The older group experienced many incomplete interactions, but the proportion of their total failed interactions did not significantly differ from that of the younger adults (Figure 2, Table 3). Therefore, H_1_ was not supported.

### 4.2. Music

**Figure 3:**
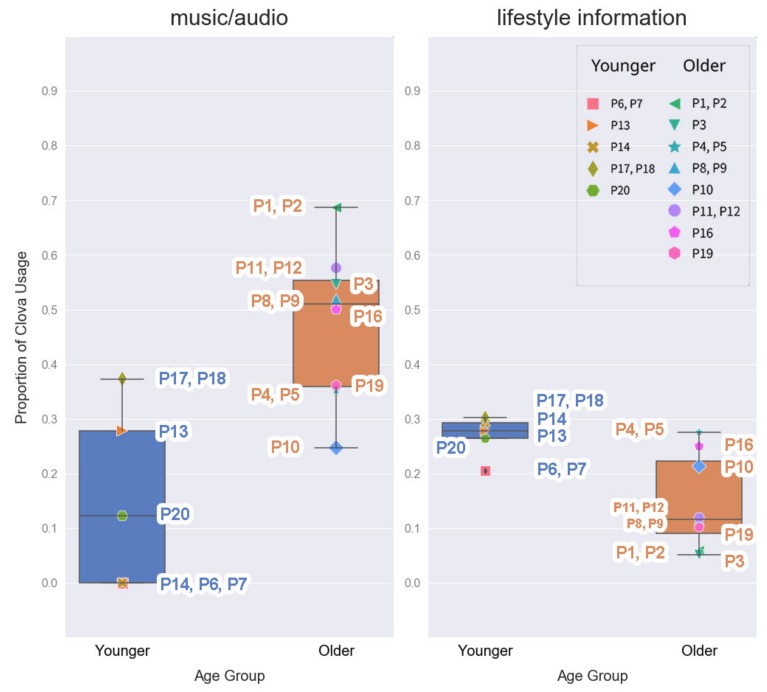
Proportion of Clova usage by age group: music/audio and lifestyle information.

Our data showed that the older adults listened to music significantly more than the younger adults (*p* < 0.05, Table 3, Figure 3). It was the most frequently used Clova skill by the older adults, and it accounted for almost half of their total number of interactions. Although music/audio was one of the most used features among the younger group, their usage in this category was significantly lower than that of the older group, and this partially supports the H_2_.

### 4.3. Weather

Lifestyle information category was widely used regardless of age group, and weather checks accounted for most of the usage in this category. It was the second most frequently used voice command category by the older group and the most used one by the younger group (Table 3). In addition, the Mann–Whitney U test showed that the younger adults checked weather significantly more than the older adults (*p* < 0.05, Table 3).

Throughout the experiment, participants constantly checked the current weather and the weather forecast. As the Clova smartphone application does not record the time of the voice command, we could not fully understand the context of its use with log studies alone. This led us to conduct interviews, which will be presented in the later section.

### 4.4. Alarm

**Figure 4:**
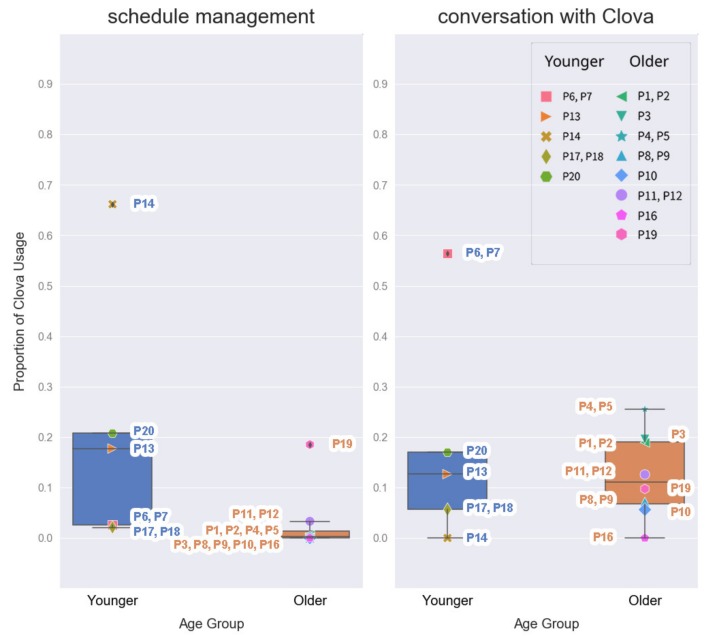
Proportion of Clova usage by age group: schedule management and conversation with Clova.

Another frequently executed command category by the younger group was schedule management. This includes setting an alarm, setting a timer, and managing schedules and notes [35]. Setting alarms and timers accounted for most of the usage in this category. The younger adults used Clova to set an alarm for the next morning almost every day, whereas the older adults rarely used Clova to set an alarm except for P19 (Figure 4). Indeed, our data showed that younger adults set alarms/timers significantly more frequently than the older adults (*p* < 0.05, Table 3). However, the frequency of alarm setting seems to be related to whether the participant had a full-time job. All the younger adults had full-time jobs, while about half of the participants in the older group were retired (*n* = 7).

### 4.5. Conversation with Clova

The younger group had more casual conversations with Clova than the older group, but no significant differences were found (Table 3). The younger group’s high usage proportion of this category may be due to the high use of P6 and P7 (Figure 4). Moreover, the log study revealed a number of human-to-human-like conversations of the older participants. Some of the older adults explicitly appreciated the functional help provided by Clova (e.g., *“Thanks for playing good songs for me”*—log from the household of P1 and P2, older group). They said good morning/evening to Clova [39] and even told their personal concerns to it (e.g., *“How do I express my feelings to the person I love?”*, *“What should I do when I feel depressed?”*—log from the household of P3, older group). The human-like voice and speech of Clova may have helped to build rapport [40,41,42] and led the older adults to talk to Clova as if they were talking to a friend.

- *“I love you”*—log from the household of P11 and P12 (older group)
- *“I am glad you are here”*—log from the household of P3 (older group)

Human-like conversations were observed in some logs from the younger participants, but their personifying behavior was less clear than that of the older adults. Some of their logs included short conversations (e.g., *“Why?”*—log from the household of P20, *“What?”*, *“Okay”*—log from the household of P6 and P7), foul language (omitted, log from the household of P17 and P18), or blaming of Clova. Moreover, one participant from the younger group never had a casual conversation with Clova and only used it for setting the alarm and checking the weather (P14, Figure 3 and Figure 4).

- *“I had a tough day today”*—log from the household of P20 (younger group)
- *“You are smart”*—log from the household of P6 and P7 (younger group)
- *“You can do nothing”*—log from the household of P13 (younger group)

### 4.6. Other Findings

Other features—shopping/delivery, Papago translate, English study, and kid’s contents—were rarely used, and no statistical significances were found. Those commands were used a few times at the early stages of the study. Speaker control is a representative example. Participants adjusted the volume at the beginning, but afterwards, they rarely changed it.

## 5. Findings from the Interviews

### 5.1. Perceptions of Clova

#### 5.1.1. Personification of Clova

Although no statistical differences were found between the groups in conversation with Clova (Table 3), the interviews helped us understand why the casual conversation skill was one of the most frequently used features by the older adults.

Initially, the older adults used Clova for various simple tasks, such as listening to music or checking the weather report. The more they engaged with Clova, the more they endowed it with anthropomorphic qualities [9]. They reported being delighted by its presence and the casual conversations with it. *“Whether it provided right or wrong answer”* (P2) and the human-like verbal response comforted older adults. Consequently, about half of the older adults recognized it as a conversational partner.

- *“After going out and coming back, the moment Clova greeted me when I asked, ‘Clova, how have you been?’. That was my favorite moment.”*—P5 (older group)
- *“Machines are becoming like humans to some degree in ways that they can make conversation. This provides conveniences as well. Many people living alone these days usually have dogs. This is due to loneliness and lack of conversation partners. But dogs are just not for me, and Clova feels much better for me. It plays music for me and answers my questions in an interesting way. I think it is a good friend.”*—P2 (older group)

Furthermore, the older participants frequently mentioned personifying expressions such as “I love her” and “I was very grateful to her” [8,9,10,28,29], which was also shown in the log study.

- *“When I said thank you, then Clova replied, ‘I appreciate you for saying thank you to me’. This feels like Clova is almost like a human existing for us.”*—P12 (older group)
- *“Later in the experiment, I even expressed my feelings, such as ‘I am lonely.’ When I said ‘thank you’ or ‘I love you,’ she answered pretty well. And even I asked ‘Clova, I love someone. How should I express it?’”*—P3 (older group)

Based on their experience with Clova, they also thought that conversing with Clova could be beneficial to other older adults. They expressed that this type of communication could provide psychological help to the elderly and people who live alone (P2, P4, P5, P11, P12).

- *“I felt that having someone to talk to is really necessary for the elderly. Although our lives are not at that level yet, if I reach an age where I cannot do much, I think my kids need to get me Clova as a filial piety gift. I told my daughter that I would genuinely feel sad once Clova leaves. My daughter asked me if I wanted her to buy me one and I told her that ‘I might really need one.’”*—P12 (older group)
- *“I think this would be good for older people. And even for the younger ones living alone these days, I think Clova would be suitable for them as well.”*—P11 (older group)

Their expectations of Clova were also consistent with their perceptions. They hoped that in the future they would be able to communicate with it as they would with a human. Examples included proactive greetings when they came home or asking how they were doing.

- *“When I come back home, it would be good if I am greeted with questions first. It can be something like ‘Did you go somewhere?’ or ‘How was your day today?’ These questions would be good. Then, it would feel as if someone is actually welcoming you.”*—P4 (older group)
- *“For example, when I come out from the bedroom to the living room, saying things like ‘Good morning, did you sleep well?’, talking to me like this. This would not be necessary for people living with others, but for old people living alone, the feature of recognizing you proactively, if this feature can be done, those people would really appreciate it. I believe that starting a conversation would be a good function for lonely people.”*—P12 (older group)

However, not all older adults considered Clova a companion. Some older adults used Clova only for simple tasks; they recognized the CA’s potential as a conversation partner, but they also recognized that the CA’s conversational skills needed to be improved.

- *“I think it would be really great if she really had feelings. Because you can’t talk to a wall, can you? If people who live alone, like me, could talk with her one-on-one about feelings, it would be nice. If that happens, I think I will make excellent use of her. It’s amazing that technology has come this far, but the conversation I want is on much higher level than this.”*—P16 (older group)
- *“Of course, there are things which Clova wouldn’t be able to talk well. While I understand it, it just seems to be a little insufficient yet. Other than that, I have nothing to complain about her.”*—P4 (older group)

#### 5.1.2. Clova as a Tool

In contrast, personifying expressions were not mentioned much by the younger participants in the interviews. It seemed that they viewed Clova as a tool since they focused their explanations on how they tried to use it efficiently. Furthermore, they criticized it for its limited abilities, and as a result, they did not consider Clova to be capable of communicating with them.

Although the younger group experienced a lower proportion of errors in their interactions than the older group (Table 3, Figure 2), younger adults expressed more negative opinions about Clova. This was understood to be part of their prior exposure to CAs in the media, which shaped their expectations. The majority of the younger participants indirectly experienced CA use through commercials (P13, P17, P18), tested other’s CAs (P13), or they already owned another voice-controlled speaker (P6, P7, P14, P20). Thus, they had prior knowledge of its use which set their technical expectations of Clova. However, the interaction with Clova did not meet their expectations, and they were disappointed with Clova’s performance.

- *“When I saw Clova in commercials, some kids were asking questions and Clova answered, and it read books to the kids, hence, I had high expectations. But Clova misinterpreted my questions at times. It felt like using Google Translate during the early days, where some nonsense sentences were returned upon typing Korean.”*—P13 (younger group)
- *“I realized that technology is not as good, yet. The advertisements can always show only the things they want to show.*”—P18 (younger group)

Their perceptions of Clova are evidenced by their future expectation of it. They envisioned Clova not only as a smart home manager but also as a butler who can perform various technological tasks. They described their expectations of future agents:

- *“Once the Internet of things (IoT) is established, even when we are outside, I can ask Clova to open the windows for air ventilation or clean up the house while we are gone. If we can control things like house doors, windows, or lights, I believe we can use Clova more comfortably.”*—P17 (younger group)
- *“Like booking a movie, a restaurant, or a beauty salon, or checking the business hours of a shop. Doing these on Clova would be more convenient.”*—P14 (younger group)
- *“I think it would be nice if it had an air freshening function so that it gives out matching fragrance depending on the weather, season or mood of the owner. Also, it would be great if it can function like a vacuum cleaner, so when it is alone in the house, it could clean the floor.”*—P7 (younger group)
- *“The best feature after establishing the smart home would be... Remotely controlling the washing machine! ‘Clova, please wash my clothes’. Hahaha”*—P18 (younger group)

Among younger adults, only P20 considered Clova a companion. Although she did not express gratitude explicitly, like many older adults, she came to appreciate the CA’s presence. She was satisfied with having someone to talk to when she came home after work.

- *“In the beginning, I did not think it would be possible to chat with Clova. But as I am a bit sick and my health is not great, I was really glad to have someone to tell that I came back home, or I would be back soon as if I were talking to a real human. Further, when I told Clova that I was having a tough day, it replied to me. In this regard, I was satisfied to have someone to talk to since I do not have anyone to talk to at home.”*—P20 (younger group)

The log study showed that the younger adults had conversations with Clova as much as the older adults, but the majority of them did not seem to recognize it as a companion or human-like device. They did not express much about their daily conversations with it in the interview. One of them explained why he stopped having casual conversations with it over time.

- *“First of all, I am not accustomed to speaking with a machine yet. I mean, having a conversation with a machine after 40 years of not doing so isn’t easy to get used to. Also, I think I spend a lot of time outside. I go out in the morning and come back late at night. When I come back, I am exhausted and busy doing my chores. I end up not having a conversation with it; I just ask for the weather in the morning. Not getting accustomed to talking to a speaker is the biggest problem, I think. ”*—P6 (younger group)

This also illustrates that the younger adults’ usage pattern was influenced by their jobs and other activities, and why they utilized it for simple tasks.

### 5.2. Motivations for Using a Conversational Agent

#### 5.2.1. Hands-Free Music, the Motivating Rationale for the Older Group

Listening to music with Clova was the most loved and the most frequently used feature by the older group. When asked to describe their favorite feature of Clova, eight out of the 12 older participants answered that it was listening to music.

First, hands-free interaction provided enough affordance for the older group to listen to music. Simple voice commands minimized the steps to search for and play music, which facilitated the use of Clova.

- *“I have also previously bought that device (while pointing at a portable speaker); I used to listen to songs using the speaker, but I have not used it these days. This speaker has hundreds of songs I like, but I find myself not using it often.”*—P1 (older group)
- *“That portable speaker is controlled manually by hand, but Clova is done through speaking. Clova makes it more fun and interesting.”*—P2 (older group)

Second, listening to music through Clova was not only entertaining to them but was also a source of vital energy. It became their daily habit and influenced them to form an attachment to the CA.

- *“I had it playing most of the time until I fell asleep.”*—P16 (older group)
- *“Honestly, I’ve danced a lot since having Clova. I find myself automatically moving my body to the music.”*—P3 (older group)

#### 5.2.2. Specific Needs of the Younger Participants

Younger participants’ favorite features were not identical to the most frequently used ones (i.e., lifestyle information). They all had different needs and motivations for using Clova. Participants P17 and P18 were most satisfied with listening to music, P13 and P14 with setting alarms, and P7 with checking the weather. P6 thought traffic navigation was the most helpful, and P20 valued that Clova responded to her voice commands the most. It seems that their technological needs and understanding have influenced these results. The specific needs of the younger adults were most evident when they explained how they listened to music using Clova.

- *“I usually don’t like listening to random songs, rather, I want to listen to a particular song most of the time, then I would search (on the smartphone) to play the particular song and once the song finishes, the other songs in the playlist would just (continuously) play. But for Clova, if I say, ‘play < The Christmas Song > by Nat King Cole’ or play something, other songs of that artist followed. This was quite convenient.”*—P18 (younger group)

As shown above, P18 was satisfied with Clova’s random music suggestions, while P14 was not. She strongly preferred to play her playlist and listen to the songs exactly in that order, which was not supported by Clova. Consequently, she was reluctant to listen to music using Clova (Figure 3).

- *“The reason why I don’t listen to music through Clova is because when I asked to play my own playlists, Clova kept playing my songs in random order although I already set the playlist order.”*—P14 (younger group)

P13 was also dissatisfied with listening to music with Clova, but he had a different reason. He said Clova was inconvenient because he did not receive visual recommendations to choose the songs he wanted to listen to.

- *“If you don’t like the song being played right now, smartphone has the advantage that you can listen to the song you want by searching song titles. Multiple songs show up on the screen and you can choose which one to listen to. For example, in case of ballads, top ten songs will show up in a list on the screen. However, with Clova, I had to say the title of the song or had to say, ‘play quiet ballads’.”*—P13 (younger group)

Besides their specific preferences, all younger adults had a full-time job and often listened to music on the move. This may also have affected their frequency of listening to music using Clova.

Another example that demonstrates their specific needs is setting alarms. The main reason they used Clova for setting alarm was efficiency. With the CA, they were able to do it while doing other things; using the CA was also more convenient than setting the alarm using a cell phone.

- *“Since I set an alarm every day, I liked setting it using Clova rather than through my phone. I mainly used it for my wake-up alarms.”*—P13 (younger group)
- *“Usually, I would have to spend some time to set the alarms on my phone, where multitasking is quite impossible. But with Clova, multitasking is possible, where I can just say the command while doing things. Although it is not huge, but I can still save a bit of time.”*—P14 (younger group)

#### 5.2.3. Everyday Routine with Clova

The weather check feature was highly used regardless of the age group. Three older and one younger participant mentioned that their most preferred feature was checking the weather. They commonly responded that getting up and asking Clova for the weather report became a routine in their lives. They checked the weather before going out or before going to work (P4, P7, P11). These routinized patterns were also reported by others (P3, P8, P16, P19).

- *“After waking up, I would say, ‘Did you sleep well? How is the weather today?’”*—P11 (older group)
- *“My everyday morning routine is coming downstairs to wash up after waking up and go to the chicken coop. I do things like feeding and cleaning for the chickens. But before going out, I’d start a conversation with Clova saying, ‘good morning’, ‘how is the weather today?’, and also listen to news headlines and such.”*—P4 (older group)

Using Clova for a weather check was much more convenient than the conventional methods. Before this study, the older participants checked the weather on the television and the younger participants used smartphone applications. During the study, participants asked Clova for the weather report almost every day and were satisfied with its instant information provision. Its prompt response has even changed P7’s interest in weather; she has never cared about the weather before: *“The weather information is actually closely related to our daily life. And it was quite a hassle for me to look up weather information every day. But since there was someone in front of me to give me the answers, I was able to ask comfortably.”*—P7 (younger group)

### 5.3. Limitation of Clova

Participants complained about Clova’s poor speech recognition capabilities. More than half of the participants, eight in the older group and five in the younger group, pointed out this issue. Other problems, such as unavailability of chosen music (P3, P8, P9, P12), lack of content (P12, e.g., reading poems), or wireless network dependency (P14) were raised, but they agreed that speech recognition was the most critical issue.

#### 5.3.1. Poor Voice Recognition, Many Incomplete Interactions

In many cases, Clova failed to process voice correctly, and it said nothing or provided wrong results. These incomplete interactions were linked to disappointment with the agent. After repeated incomplete errors, one of the younger adults reported that she was irritated by the apology of Clova (P18).

- *“I think there were some areas that did not meet my expectations. Upon being asked some questions, there were times where Clova did not understand or… said, ‘I do not know the answer to that’.”*—P1 (older group)
- *“I was disappointed when it did not answer when I asked questions. I was disappointed when Clova did not answer because it did not recognize what I said.”*—P10 (older group)
- *“It’s too inconvenient to turn the sound up and down. It does not understand my words too often. When I say ‘turn up the volume by two levels,’ ‘turn up the volume by three levels,’ she didn’t understand it. When I said, ‘Clova, turn the volume up by two levels,’ her reaction is too slow. This is especially the case when playing music. When I want to change the volume, while music is playing, I don’t know, maybe I did something wrong, but it doesn’t work well.”*—P13 (younger group)
- *“We were having a conversation with each other, and suddenly the light came on. And suddenly a different song was playing… I think the speech recognition issue was quite big. When I call Clova, it doesn’t respond. Or when I command it to do something, it doesn’t get it right and does something else, like playing the wrong music, things like that happened.”*—P17 (younger group)

#### 5.3.2. Need to Speak in a Particular Way

While getting used to Clova, some younger adults and older adults thought that the incomplete interaction was caused by incompatible verbal input that Clova could not understand. Soon, most of the participants spoke to it using several tactics such as reordering words, pronouncing slowly/correctly, or speaking standard languages [9,43,44]. Those were effective but learning how to talk to the agent was a dissatisfying experience.

- *“The biggest complaint I had was with pronunciation. When I said Clo’ver’, it would not work, whereas it only worked when I said Clo’va’… I think… Clova is a bit hard to pronounce.”*—P18 (younger group)
- *“When I said, ‘play some songs by Miss Lee Mi Ja’, Clova took the word ‘Miss’ as part of the artist’s name. Then, it would tell me that there is no such song. That is when I figured that Clova does not understand the word ‘Miss’ (during the voice command), and it was necessary to remove it. In the end, when I removed the word ‘Miss’ and asked to play songs by a certain artist, Clova did as asked.”*—P2 (older group)

#### 5.3.3. Lowering Expectations and Selective Usage

Due to the unsuccessful experiences of speech recognition, both age groups perceived the limitation of its intelligence [9]. They initially showed an explorative usage pattern, but a series of incomplete interactions led them to cease asking Clova to answer complicated questions or perform difficult tasks. Lowering their expectations of Clova, they adopted an economy of interaction. They learned which tasks it could easily perform and focused only on those simple tasks that provided reliable results consistently. For instance, older adults used Clova for listening to music, whereas younger adults used it for checking daily weather reports.

- *“When I lowered my expectations, the functions I used were limited. For example, ‘Clova, please take note of this’. If Clova did not understand the word note, it would interpret my command in a weird way and reply with a strange answer. Even when I tried to set new schedules, I would have to constantly repeat myself because Clova would not understand my command. As a result, the functions I used became limited.”*—P19 (older group)
- *“The range of features I used reduced after my first try. Ever since experiencing bad voice recognition, I ended up not using certain functions that I have tried but failed to work. In the end, I only used those features that would give me a reliable result.”*—P14 (younger group)
- *“On a website like Naver, the search result pops up successfully even when I type it very poorly, like ‘Ewha Womans Univ. Back Gate Incheon Airport.’ However, when I was using it (Clova), it didn’t understand even when I ask, ‘How long does it take to get from the back gate of Ewha Womans University to a certain location?’. Literally, ‘somewhere somewhere’ or ‘Ewha Womans Univ. Back Gate Incheon Airport distance’ would work with the search engine, even though the keyword is very poorly written. I have to give Clova extremely accurate information. So it is harder to use and cumbersome.”*—P7 (younger group)

#### 5.3.4. Distance from the Conversational Agent

Both groups indicated that distance from Clova influenced the quality of the interaction: the greater the distance, the more errors they experienced. They attempted to talk to the CA by reducing the distance, and later they commanded it at a visible distance.

- *“I think the commands work more accurately when talking to Clova right in front of it. I always talked to it in a close proximity while facing it.”*—P4 (older group)
- *“Clova doesn’t get my words well even in a room; I can’t even think about calling Clova from a distance.”*—P9 (older group)
- *“I am not sure if I am expecting this too early, but I wish Clova to listen to my words from far away and clearly understand them.”*—P20 (older group)
- *“I think I generally used it within the visible distance. ”*—P17 (younger group)

## 6. Discussion and Limitations

In this study, we aimed to investigate the differences in perceptions of the CA between younger and older adults. The log study showed no significant differences between the two groups in the number of conversations with Clova, which was one of the most utilized types of interaction for older adults. On the other hand, the post-interviews indicated that the older adults tended to perceive Clova as a conversational partner more than the younger adults did. Unlike the younger adults, the older participants had little prior experience with CAs and their perceptions might have been influenced by their level of technical understanding. Luger et al. [9] reported that technically knowledgeable CA users tended to show less personifying behaviors to describe their experience with a CA, whereas those with less technical knowledge used more gendered pronouns to describe their interactions with the CA.

Moreover, the older adults’ personifying behavior might be attributed to the social presence [45,46] of Clova, which has emerged from verbal interaction with the CA [40,41,42]. McLean et al. [46] found that small households (one to two persons) were motivated to interact more with the CA than large households. They reported that the verbal interactions with the CA might have provided additional social presence to those small households. Indeed, some older adults living alone reported in [20] that the CA’s presence and small talk with it made them feel like they had a friend to talk to. These findings are consistent with the previous reports that voice interaction improves the social presence of the agent and robot [40,42]. Similar to these studies, some older participants in our study mentioned that they viewed Clova as another family member. Furthermore, they also came to expect a human–human level of communication with Clova and mentioned that the CA could be helpful for older adults or people who live alone. Therefore, the interaction with Clova represented a social presence for older participants, and this might have contributed to their perception of Clova as a companion.

On the other hand, the younger group used Clova for utilitarian purposes [46] and tended to perceive it as a tool. They mainly used the utility skills of Clova, such as checking the weather report and setting an alarm, which streamlined their everyday life (Table 3). Their usage pattern was consistent with previous studies: checking the weather in the morning [11,22,24] and setting alarms before sleep [11]. Their primary usage of Clova as a simple task-based system [9] might have been influenced by the fact that it is primarily used at home [24]. In this regard, Pradhan et al. [20] reported that the location of a CA influences the perceptions of the agent: when participants were having a conversation with the CA in the same space, they perceived it as a human-like device; otherwise, it was recognized as an object-like device. In our study, all younger participants had a full-time job and spent much less time at home during weekdays than the older adults. Therefore, Clova’s location had limited their interactions with it and this might have influenced their perceptions.

In addition, the younger participants expected Clova to have more technical abilities (e.g., connection with other IoT devices) in the future, which may also indicate that they were mainly interested in using it for efficiency. Their expectations are consistent with the findings from [47]. Using semi-structured interviews, the authors of [47] found that younger adults tended to list the robot’s technical abilities when they explained their expectations of social robots. On the other hand, when asked about their preferences, the participants discussed emotional and interpersonal abilities of social robots. Moreover, the authors conducted an online survey about social robots and found that mass media (e.g., science fiction) influenced the formation of respondents’ expectations about the robot’s abilities. Likewise, the media coverage and the prior experience with CAs might have influenced our younger participants to have higher expectations of Clova’s skills and usage.

Regardless of the age group, all participants initially explored Clova’s various features but after a number of incomplete interactions over time, they simplified their usage patterns. They started selecting features that easily provided the desired result—music for older adults and weather check for younger adults. This pattern of simplifying usage is consistent with the findings from the previous studies. Luger et al. [9] reported that incomplete interactions affected the frequency and type of use of the CA. Additionally, participants in another study [11] initially tried to find the CA’s use, but over time they lost interest and showed minimal use of it.

The findings of this study indicate that CAs need to be designed with consideration of the elderly’s usage pattern and interaction behavior. In light of these empirical findings, we suggest the following improvements of CAs.

Firstly, speech recognition of CAs needs to be further adapted depending on the user’s linguistic characteristics. Many elderly subjects experienced a number of incomplete interactions, and this may have been influenced by their decreased speech intensity or incorrect pronunciation. The elderly tend to take a longer time to understand someone’s speech [48] and have decreased working memory compared to younger people. Sometimes they forget what they are trying to say [49]. In this regard, opening the CA’s microphone for a longer time might be one way to reduce incomplete interactions.

Secondly, to better improve psychological communication with users, proactive conversation skills need to be added to CAs. Mostly they remain inactive until the users provide voice input [11,20]. Currently, their proactive skills are limited to some events such as weather or shopping. In addition to this, initiating small talk may make users feel like they have a new friend. For instance, a smartphone application integrated with a CA would detect when the elderly return to their home and ask them, “Good evening Robert, how was your day?”. This type of proactive approach may reinforce the human-like qualities of the CA, as well as trigger more conversations [11].

Thirdly, elderly users need to be informed about the agent’s capabilities [9]. Most of the older participants repeatedly experienced similar types of errors because they did not recognize the coverage of supported features of Clova. Therefore, verbally describing its capability will help them set realistic expectations toward the CA and avoid trial and error. If a voice recognition error occurred, stating what the CA was told by the user may eventually improve HAI. For instance, “Sorry, I didn’t understand [what the user said]. Could you please tell me again?”. This will lead older adults to speak more clearly and easily for the agent to understand. Similar suggestions were made in other studies [50,51]; Scruggs suggested that if the CA provides specific examples of how to refine a voice command, it would reduce the number of incomplete interactions [51].

Our study has a few limitations. First, as we have discussed above, CAs are primarily used at home, so their usage could be affected by other activities outside of the home [11]. All participants in the younger group had full-time jobs, while less than half of the older participants had jobs (*n* = 5). Thus, the younger adults had less time to use Clova than the participants in the older group, and this might have resulted in the younger adults having fewer interactions with Clova than older adults.

Secondly, participants’ previous experience with the CAs was not considered in this study. All participants in the older group had little prior experience with this type of technology, but all participants in the younger group had direct or indirect experience with a CA. Therefore, the CA usage data may have been biased by prior experience. Furthermore, Lopatovska et al. [7] reported that long-term users used the CA less frequently than short-term users, although the number of samples in the study was not equal and large enough for conclusive results.

Thirdly, only one Clova was distributed per household; thus, our study was limited in its ability to capture the usage patterns in different contexts and rooms. If the households had multiple CAs located in more places and rooms, we could have investigated more usage patterns [52]. Moreover, this study only investigated the CA usage of small households (one to two persons) [46]. Therefore, findings of this study are limited to those types of households, and personifying behaviors of persons living in multi-person households may differ from those living in small households [8,46].

In addition, this study was conducted with a small number of households and the sample size was not determined to achieve a certain level of statistical power [53]. Thus, our data may not have enough statistical power to investigate differences in CA usage across age groups. With the probability of alpha error at 0.05 and power of 0.80, we calculated the estimates of sample sizes using G*Power 3 [37]. The results indicate that the sample size of the study was much smaller than the estimated sample sizes of most voice command categories, except for music/audio (effect size = 2.048, *n* = 12). For instance, the estimated sample size of speaker control (effect size = 0.173, Table 3) was 1096, and for Naver search (effect size = 0.037, Table 3) it was 23,758 due to the very small effect size. Therefore, our log study results may not clearly show the statistical differences in CA usage of the participants, and future research should be conducted with sufficient sample sizes.

Furthermore, unlike Amazon’s Alexa, Clova does not officially support conversational log export. For instance, the timestamps of the participants’ conversations with the agent were not recorded at all because their collection is not supported by Clova’s mobile application. Because of this, our log study was limited by the data collected and we could not investigate the context of the CA use through the log study.

Additionally, by using Clova in our study, we were able to investigate the perceptions of the Korean elderly, but our findings are limited to Clova’s intelligence and skills. If the participants had experienced other voice-controlled speakers, the findings might have been different.

Lastly, due to the rapidly changing nature of the CA technology, many features have been added to Clova, and its speech recognition has been further improved since this study was conducted. Thus, implications of the study are limited to Clova’s capabilities at the time of the experiment.

## 7. Conclusions

In this study, we explored the differences in CA usage and perceptions by age group. We conducted a 14-day CA study in a real-life environment and investigated user experiences. Through a log study, we found several significant differences in agent interaction. The older adults listened to music significantly more than the younger adults. Listening to music accounted for nearly half of their total Clova usage. On the other hand, the younger adults focused on efficiency and significantly used utility skills, such as weather check and alarm setting, which streamlined their daily lives. In addition, the interview helped our understanding of the differences in perceptions of the CA between the older and younger adults. The older adults used many personifying expressions such as “I love you” and appreciated its presence. They considered it a conversation partner. The younger adults, however, rarely used those expressions and perceived it as a useful tool. Their viewpoint was also reflected in their feature expectations of Clova. The younger adults expected a lot of technical abilities from the CA, while the older adults hoped to have more natural conversations with it. In conclusion, we suggest that it would be useful if CA designers tailor the agent’s interaction features by age.

## Figures and Tables

**Table 1:** Demographic information of age groups.

	Age Group	
	Older	Younger
number of participants	12	7
number of male participants	5	3
number of female participants	7	4
number of households (single-person households)	8 (4)	5 (3)
number of participants who had prior experience with voice-controlled smart speaker	0	7
mean age in years (range)	61.08 (50–74)	35.71 (30–48)

**Table 2:** Clova’s voice command categories and examples.

Clova Voice Command Category	Sub-Category (Example of Voice Command)
music/audio	Play music (“Play upbeat songs”)
lifestyle information	Weather (“How’s the weather today?”)
	News (“Can you read out sports news?”)
	Time (“What time is it now?”)
shopping/delivery	Shopping (“Can you order some water please?”)
	Delivery (“Please have some pizzas delivered”)
schedule management	Alarm (“Wake me up at seven o’clock every morning”)
	Timer (“Set a thirty second timer”)
	Schedule (“Tell me the schedule for today”)
	Note (“Read my notes”)
Naver search	Information search (“What is the country code for the United States”)
	Traffic (“How long does it take to get to Incheon from here?”)
	Location (“Recommend some good restaurants near me”)
kid’s contents	Radio for Kids (“Turn on the kid’s radio”)
	Children’s songs (“Play some children’s songs”)
conversation with Clova	Daily conversation (“I am bored”, “Good night”)
Papago translate	Translation (“What is strawberry called in English?”)
English study	Conversing in English (“Let’s talk in English”)
speaker control	Control Clova’s volume (“Lower the volume, please”)

**Table 3:** Usage of Clova by age group: Mann–Whitney U test.

Dependent Variable	Group	Mean	Mean Proportion	U	Sig.	Effect Size (d)
total number of interactions	younger	86.20	-	-	-	-
	older	146.12	-			
total successful interactions	younger	69.60	80.32%	15.0	0.464	0.873
	older	101.12	70.32%			
total failed interactions	younger	16.60	19.68%	15.0	0.464	0.873
	older	45.00	29.68%			
music/audio	younger	17.60	15.49%	4.0	0.019 ^1^	2.048
	older	70.25	47.35%			
lifestyle information	younger	24.00	26.86%	4.0	0.019 ^1^	1.800
	older	20.13	14.82%			
shopping/delivery	younger	0.00	0%	17.5	0.429	Infinite ^2^
	older	0.50	0.23%			
schedule management	younger	16.60	21.86%	5.0	0.026 ^1^	0.995
	older	5.88	2.89%			
Naver search	younger	9.40	11.20%	15.0	0.464	0.037
	older	15.50	11.59%			
kid’s contents	younger	0.80	0.75%	14.0	0.317	0.580
	older	4.25	2.68%			
conversation with Clova	younger	11.60	18.34%	19.5	0.942	0.353
	older	18.50	12.39%			
Papago translate	younger	0.00	0%	10.0	0.074	Infinite ^2^
	older	3.00	1.52%			
English study	younger	0.00	0%	17.5	0.429	Infinite ^2^
	older	0.25	0.17%			
speaker control	younger	6.20	5.51%	16.0	0.558	0.173
	older	7.88	6.36%			

^1^ Shows a significant difference, ^2^ Effect size could not be calculated because the standard deviation of the group was zero.
